# Analysis of urban necessities reserve index and reserve quantity under emergency conditions

**DOI:** 10.3389/fpubh.2024.1402998

**Published:** 2024-07-31

**Authors:** Qijun Jiang, Xiaoyang Ji, Zhijie Rong

**Affiliations:** School of Economics and Management, Shanghai Ocean University, Shanghai, China

**Keywords:** necessities of life, reserve index, reserve quantity, emergency reserve, city

## Abstract

While maintaining a robust reserve of daily necessities is crucial for urban safety, but there is a lack of scientific basis for determining “what to store” and “how much to store.” This paper address this gap by classifying and summarizing the emergency materials of urban necessities in Shanghai, and establishing a corresponding reserve list. By constructing an index model of daily necessities reserve, this paper provides a scientific foundation for “what to store.” Additionally, the reserve levels of different types of daily necessities are classified and managed, the reserve model of emergency daily necessities is constructed. This approach clarifies the scientific basis for “how much to store,” overcoming the problems of subjective factors interference and the potential mismatch between the results of objective weighting method and reality. Furthermore, to better cope with emergencies, countermeasures and suggestions are put forward: optimizing the material structure of emergency reserves, managing the material reserves at different levels, scientifically and reasonably planning the amount of emergency materials, and reducing the cost of reserves and improve the efficiency of emergency reserves.

## 1 Introduction

Global warming intensifies the instability of the climate system, with frequent extreme cold and warm events, and frequent droughts and rainstorms becoming a new normal (1). This undescores the importance of constructing an urban necessities reserve system. Under the emergency situations of natural disasters, wars, public health incidents, accidents, and other serious disasters, such system is crucial for urban residents to maintain their normal lives, and it is also an important micro-embodiment of urban resilience (2). In recent years, the State Council has repeatedly proposed to improve the reserve mechanism of residents’ daily necessities in relevant documents concerning the circulation industry, emergency system, and economic system improvement (3). The efficient and accurate management of daily necessities in emergencies is a systematic work that requires considering many factors in the management process, and the storage requirements and conditions of each type of material are different (4). After the outbreak, megacities consolidated and optimized the experience model of epidemic prevention and supply, and many cities incorporated emergency supply of daily necessities into the smart governance of megacities and the construction of living materials support capacity in the strategic rear area according to the idea of “wartime supply, peacetime regulation, peacetime combination, and agile switching” (5).

To improve the emergency support ability of daily necessities in emergencies such as public health emergencies, we can focus on four key areas: technology, management, storage, and logistics. First, we can rely on scientific and technological means to achieve efficiency emergency management of daily necessities. This includes establishing a scientific and technological support system and building an integrated emergency support platform for daily necessities (6). Second, it is necessary to improve the emergency material support mechanism. This involves creating a coordinated support system, identifying and evaluate risks (7), and establishing relevant management organizations to provide emergency support for the government’s emergency material allocation management (8). Third, we should also strengthen research and application of monitoring technology for emergency storage channels of daily necessities (9). We should further improve the storage system of daily necessities and establish a scientific material storage system (10). Fourth, building a robust emergency logistics system is essential. This system should encompass a command system, channel system, information management system, facilities and equipment system, and security system (11). At present, China has established a relatively well-developed urban reserve management system, and the main challenge is the reserve of daily necessities. However, the scope of daily necessities evolves with societal development, and reserve and allocation in sudden disasters constantly face new challenges (12). Under the emergency situations, the city’s regular resource allocation system becomes easily disrupted. Determine the categories, storage quantities, and storage methods of daily necessities (13), and identifying approaches to ensure the supply of urban daily necessities, are urgent issues that need to be addressed.

The type, quality, and speed of the supply of necessities, as well as whether it can meet people’s needs in a timely manner, can indirectly reflect the local government’s ability to guarantee emergency response. Necessities of life are items that can satisfy the most basic needs of people’s survival and life. Although market serves as the primary supplier of necessities goods, during emergencies, local governments play a crucial role in emergency management by issuing notices related to the supply of necessities of life, and providing green channels and policy concessions for the relevant enterprises, to ensure that the supply of essential commodities meets public needs. While the National Classification and Coding of Emergency Materials and the Catalogue of Emergency Support Key Materials provide a clear description of emergency materials, there is no well-defined judgment basis for the confirmation standards of reserve varieties and reserve quantities of urban necessities in academic circles and industries. For the reserve of daily necessities, simply having more reserves is not necessarily better. Effectively improving the reserve efficiency of urban daily necessities is an important part of reserve management. This paper deepens the research on how government can guarantee necessities of life during emergencies such as public health emergencies. Existing research on public health emergencies in China focus on the exploration of the causes and the construction of the system, with less emphasis on the effective emergency reserve management under such events. At the same time, although the stockpiling of daily necessities has been strongly advocated, there are no clear regulations on the quantity and types of daily necessities to be stockpiled in cities. Taking Shanghai as an example, this paper analyzes the reserve index and reserve quantity of various types of daily necessities based on the risk characteristics, environmental characteristics and demand characteristics of daily necessities in Shanghai, comprehensive risk probability, urban population characteristics and other factors. This analysis clarifies the importance of different types of daily necessities reserves, improves the distribution method of daily necessities in emergencies, promotes the construction of the daily necessities reserve theory in emergencies that aligns with China’s national conditions and guides practical implementation, enriches the contents of emergency support such as public health, and has significance for effectively guiding the hierarchical management of daily necessities reserves, thereby improving urban residents’ livelihood security.

## 2 Literature review

### 2.1 Definition and classification of daily necessities

In response to emergencies, the government stockpiles a certain amount of relief materials to ensure the supply of urban necessities (14), especially in case of inconvenient transportation, such as the closure of the city caused by emergencies and the shortage of residents’ daily necessities. Local departments must then arrange and deploy resources in a timely manner to ensure the basic needs of the residents who during city closures. The necessities of life are the basic living aid materials used to ensure the emergency transfer and resettlement of personnel and meet their basic living needs in emergencies. The public’s understanding of the word “necessities” can vary (15), and personal views are influenced by individual circumstances, making it difficult to reach a consensus on the definition of necessities that are completely public-oriented (16). In a broad sense, necessities of life cover the protection of food, clothing, housing, transportation, medical care, sanitation, and other aspects, including but not limited to five functional categories: temporary residence, bedding, clothing, food, medical and epidemic prevention. From an economic point of view, the demand for daily necessities are mostly inelastic, such as food and medical services (17). In other words, even during economic fluctuations, the demand for these items remains relatively constant. From the perspective of daily life, it also includes items such as sanitary cleaning products that can meet the most basic needs of people’s survival and life. In 2011, Article 27 of the Emergency Management Measures for Market Supply of Necessities by the Ministry of Commerce proposed that “in the event of public health incidents which are easy to spread, such as mass diseases and animal epidemics, it is necessary to focus on the market supply of sanitary and cleaning products, protective products, grain, edible oil, salt, livestock and poultry products, and convenience foods” (18). Combining this information with the list of daily necessities outlined in the Tenth Five-Year Plan for Emergency Material Support, Shanghai List of Suggestions for Family Emergency Material Reserve and Guidelines for Disaster Relief Material Reserve Standards (19), this paper classifies the daily necessities into “clothing support, food support, temporary accommodation, sanitary products, medical drugs” and other varieties. The clothing support category includes regular clothes and warm clothes; Food security mainly includes drinking water, finished grain, convenience food, edible oil, edible salt, green leafy vegetables, radishes, potatoes, edible fungi, fruits, livestock meat, aquatic products, eggs, milk powder and liquid milk. Temporary accommodation mainly includes tents, movable tables and chairs, folding beds, bedding, pillows and moisture-proof mats. Hygienic articles can be divided into cleaning and disinfection articles such as disinfectant alcohol, disinfectant and insecticide, daily necessities such as towels, toothbrushes, toothpaste, roll paper and garbage bags, and sanitary protection articles such as simple toilets, bath cars and garbage bins. Medical drugs are mainly emergency medical kits.

### 2.2 Differences in demand for daily necessities under different emergency levels

The State Emergency Management Bureau divides emergencies into four grades: I (particularly serious), II (serious), III (major), and IV (general). The emergency response is divided into four levels from high to low: I, II, III, and IV (20). Level IV refers to general public health emergencies, public health emergencies with more than 10 casualties and less than 29, with more than one case of death and critical illness, and other events jeopardizing the safety of public life, which are also reported to the people’s government of the prefecture-level administrative regions. Level III refers to major public health emergencies, public health emergencies with more than 30 casualties and less than 49 casualties, more than 3 cases of death and critical illness, and other events jeopardizing the safety of public life, also reported to the People’s Government of the prefecture-level administrative regions. Level II refers to major public health emergencies, cross-city (prefecture) public emergencies with serious casualties, and other events requiring emergency medical and health care rescue, and is guided by an expert group sent by the provincial government. Level I refers to particularly significant public health emergencies, public emergencies with particularly serious casualties across provinces (districts and municipalities), and other events requiring medical and health emergency rescue, with the State Council sending an expert steering group (21). Taking the sudden flood disaster as an example (22), the level of impact on daily life varies depending on the emergency response level. When the level IV emergency response occurs, the daily life of most residents are minimally affected, and it may only increase the purchase of main and non-staple foods such as meat and vegetables. When Class III and Class II emergency responses occur, there may be more than dozens of casualties, leading to changes in demand of daily necessities. On the one hand, it is reflected in the increase of food reserves of residents’ families; on the other hand, it may be reflected in the increase of medication purchases due to the occurrence of casualties. When the level I emergency response occurs, not only casualties may occur, but also houses may collapse and people’s production and living materials suffer huge losses. This level of emergency triggers the need for temporary necessities such as clothing, food, temporary accommodation, medical drugs, and sanitary products.

## 3 Materials and methods

### 3.1 Model construction of single variety reserve index of daily necessities

#### 3.1.1 Screening standard of daily necessities reserve

Due to the wide variety of daily necessities, it is impractical to reserve all of them. In order to comprehensively evaluate the importance and necessity of the reserve of each item of daily necessities, it is essential to thoroughly analyse the characteristics of daily necessities, reserve costs, and other factors. By constructing the single variety reserve index of emergency daily necessities, we can scientifically answer the question of “what to store.” This will allow us to optimize the category composition and allocation of daily necessities reserve, resulting in reduced costs and improved efficiency of emergency daily necessities reserves. The emergency necessities single product reserve index refers to the importance of maintain this particular product in daily necessities reserve. The higher the value, the more essential it is to have a sufficient stock of this particular product.

In recent years, Shanghai, Beijing, and Guangzhou have proposed the concept of establishing a 15-min life circle (23). The typical size of a 15-min community life circle generally ranges from 3 km^2^, with a permanent population of 50,000 to 100,000 people and a population density of 10,000 to 30,000 people/km^2^ (24). To understand the standard of the daily necessity reserve, we take the 15-min walking range as the spatial scale, and allocate various functions and facilities required by residents’ basic life (25). Daily necessities are the most needed materials for residents to deal with emergencies, and also the basic reserve materials to meet the needs of residents’ 15-min living circle. After an emergency, the emergency reserve of daily necessities can meet the basic needs of the victims. Therefore, meeting consumers’ consumption demand is the most important of the screening criteria.

When planning emergency supplies, it’s crucial to consider the unique challenges of emergencies and logistics. This means finding a balance between shelf life and convenient circulation of goods. To address these, it is necessary to implement a multi-item storage strategy, to meet the overall nutritional needs of the population while maintaining convenient circulation with a relatively small amount of single items (26). In addition, given the unexpected nature of the emergency and unknown duration, it is also necessary to take into account goods that have low storage requirements and extended shelf lives.

To sum up, based on the premise of “satisfying consumers’ needs, diversifying commodity categories and facilitating storage,” to meet the basic reserve material demand of residents’ 15-min living circle and improve the energy efficiency of daily necessities reserve, this paper puts forward the following screening criteria for the selection of daily necessities single product reserve: the selected emergency materials of daily necessities must be in the list of Emergency Materials Classification and Coding, and the reserve scope should cover four aspects: eating, wearing, living and using, which can meet the requirements. The reserve of daily necessities should be closely related to the needs of life, which is universal. The chosen items should be in high demand during disasters when many residents require them. To improve the reserve efficiency, it is necessary to give priority to the economical varieties, comprehensive in function, convenient to use, and easy to reserve (27).

#### 3.1.2 Analysis of factors affecting single variety reserve index

When a disaster occurs, emergency managers should first consider whether there are available reserve materials. If there are, it will be regarded as a direct acquisition of the materials; if not, it will be considered as internal acquisition and external acquisition of the materials. The main way of internal acquisition is direct production. In case of emergency, the most important consideration is whether the material can be obtained through production in a timely manner. If the materials can be produced quickly during an emergency, they are considered to be available. Conversely, slow production necessitates reserve stockpiling. The primary factor for external acquisition or purchase is the market liquidity of the material. Materials with strong market liquidity are considered to be available. If the liquidity is not strong, the rational decision is to reserve the material for a rainy day. To sum up, when it is difficult to obtain a material internally and externally, the rational decision is to reserve it, and the storage cost and storage resistance of this material will further affect the storage strategy. Therefore, this study identifies four key factors for screening the single variety reserve index of daily necessities: market liquidity, production cycle, storage resistance, and storage cost of materials.

Market liquidity of reserve items. Market liquidity is the possibility and speed of market participants to reach a transaction at market price. Commercial reserves usually reserve emergency materials and equipment with large market liquidity, high storage costs, and short shelf life (28). The higher the market liquidity of materials, the easier it is for residents to buy such materials in case of emergency. Therefore, the influence of market liquidity on the reserves of such materials is negative.

Production cycle of reserved items. When an emergency happens, the demand for living materials has the characteristics of timeliness, suddenness, and universality. Therefore, when formulating the emergency material reserve strategy, the length of the production cycle of the material should be considered. Emergency materials with a long production cycle are difficult to produce quickly in response to an emergency (29), necessitating a larger reserve. Therefore, the impact of the production cycle on the reserve of such materials is positive. In the context of emergency procurement, emergency materials with large demand, short production cycles, and easy preservation are usually studied (30).

Storage resistance of reserved items. Storage resistance refers to the characteristics that materials can maintain their original quality without obvious adverse changes within a certain storage period. When considering the storage capacity of a single item, the storability of the single item is often considered to reduce the inventory cost of emergency materials and the waste caused by deterioration (31). The higher the storability of materials, the lower the risk of loss caused by storage time, and the influence of storability on the storage capacity of such materials is positive.

The reserve cost of a single item. During the storage of materials, there may be various storage expenses such as warehouse expenses, insurance expenses, inventory damage, and deterioration losses. The reserve cost may lead to the problem of overstock of inventory and capital occupation. If the materials are excessively reserved, it will increase the material reserve cost and lead to the waste of material resources. For example, to reduce the impact of disasters, the government reserves emergency materials to improve the efficiency of disaster relief, but it may not be able to meet the demand for emergency materials for unconventional emergencies due to factors such as reserve costs and management costs (32). Therefore, to improve the reserve efficiency, materials with high reserve costs need to be appropriately reduced.

#### 3.1.3 Calculation of single variety reserve index of daily necessities

According to the above analysis, this paper comprehensively considers four factors of emergency necessities: market liquidity $\partial_{i}$ , storage resistance $\beta_{i}$ , production cycle $\gamma_{i}$ , and reserve cost $\theta_{i}$ . The subjective and objective combination method of the Delphi method and entropy weight method is used to calculate the weight of each index, which solves the problem that the subjective weighting method is too subjective and the objective weighting method is quite different from the actual situation. The data sources are the China Logistics Association Report, China Statistical Yearbook, China Retail Yearbook, and China Agricultural Statistical Yearbook. The implementation effect of each influencing factor index is evaluated, and the initial data set of each index is finally obtained.

Because the storage capacity and production cycle have a positive impact on the reserve, the $\beta_{i}$ and $\gamma_{i}$ are positively standardized by Eq. (1). The influence of market liquidity and reserve cost on the reserve is negative, and $\partial_{i}$ and $\theta_{i}$ are inversely standardized by Eq. (2) to get the standardized index data set.


(1) $$X_{i}^{\prime }=\frac{X_{i}-\min\left(X_{1}，X_{2},\;\ldots \;\ldots ，X_{i}\right)}{\max\left(X_{1}，X_{2},\;\ldots \;\ldots ，X_{i}\right)-\min\left(X_{1}，X_{2},\;\ldots \;\ldots ，X_{i}\right)}$$

(2) $$X_{i}^{\prime }=\frac{\max\left(X_{1}，X_{2},\;\ldots \;\ldots ，X_{i}\right)-X_{i}}{\max\left(X_{1}，X_{2},\;\ldots \;\ldots ，X_{i}\right)-\min\left(X_{1}，X_{2},\;\ldots \;\ldots ，X_{i}\right)}$$

The specific steps are as follows:

For n indicators and m individual items, the value of the ith individual item under the kth indicator is the weight of that indicator: $P_{ik}=\frac{x_{ik}^{\prime }}{\sum_{i=1}^{m}x_{ik}^{\prime }},\left(i=1,2\;\ldots ,\;m;\;k=1,2,\;\ldots \;n\right)$
Entropy value of the four evaluation indicators.

The entropy values of four evaluation indexes, such as market liquidity $\partial_{i}$ , storage resistance $\beta_{i}$ , production cycle $\gamma_{i}$ and reserve cost $\theta_{i}$ , are obtained by using the equation $e_{k}=-{\left(lnm\right)}^{-1}\overset{m}{\underset{i=1}{\sum }}p_{ik}\ln p_{ik}$ .

The entropy weight of four evaluation indexes, such as market liquidity $\partial_{i}$ , storage resistance $\beta_{i}$ , production cycle $\gamma_{i}$ and reserve cost $\theta_{i}$ , is obtained by using the equation $\omega_{k}$ = $\frac{1-e_{k}}{k-\sum_{k=1}^{n}e_{k}}$ .

Substituting the standardized index data into the above equation, the entropy weights of market liquidity $\partial_{i}$ , storage resistance $\beta_{i}$ , production cycle $\gamma_{i}$ and reserve cost $\theta_{i}$ are 0.21, 0.11, 0.46, and 0.21, respectively.

Considering the market liquidity $\partial_{i}$ , storage resistance $\beta_{i}$ , production cycle $\gamma_{i}$ and reserve cost $\theta_{i}$ of emergency daily necessities, the single product storage index of daily necessities is calculated, as shown Eq. (3).


(3) $$\sigma_{i}=k_{\partial i}\partial_{i}+k_{\beta i}\beta_{i}^{\prime }+k_{\gamma i}\gamma_{i}^{\prime }+k_{\theta i}\theta_{i}^{\prime }+k_{\mu i}\mu_{i}^{\prime }$$

Among them, $k_{\partial i}$ , $k_{\beta i}$ , $k_{\gamma i}$ , $k_{\theta i}$ , $k_{\mu i}$ respectively represent the entropy weight of liquidity, storage resistance, production cycle, and reserve cost. Finally, the single variety reserve index of emergency materials is obtained as shown in Table 1.

**Table 1:** Emergency daily necessities single product reserve index.

Category	Subdivision category	Reserve index	Reserve index ranking	Average index
Clothing security category	Daily wear	0.30	13	0.290
	Warm clothing	0.28	14	
Food security category	Processed food	0.40	6	0.274
	Instant food	0.35	10	
	Bottled drinking water	0.40	5	
	Edible oil	0.24	19	
	Salt	0.34	11	
	Leafy greens and other storage-intolerant vegetables	0.18	22	
	Storage-resistant vegetables such as turnips and potatoes	0.39	7	
	Mushrooms	0.18	21	
	Fruits	0.11	23	
	Livestock meat	0.24	18	
	Aquatic products	0.23	20	
	Eggs	0.24	17	
	Liquid milk	0.26	16	
	Milk powder	0.30	12	
Temporary accommodation category	12 m^2^ single tent	1.00	1	0.584
	Bedding (bedding, pillows, moisture-proof mats, etc.)	0.83	4	
	Mobile tables and chairs	0.89	3	
Hygiene products	Cleaning and disinfecting products	0.38	8	0.467
	Daily life supplies	0.38	8	
	Hygiene and security supplies	0.97	2	
Medical drugs	First aid medical kits	0.268	15	0.268

It can be seen from Table 1 that the reserve index of temporary accommodation and office necessities such as tents, movable tables, and chairs, health protection articles, and bedding articles is the highest. During serious disasters, basic living facilities such as houses may be seriously damaged, and emergency materials such as emergency tents, quilts, and pillows can provide temporary accommodation and office facilities for victims and staff who carry out the rescue. Moreover, temporary accommodation materials such as emergency tents, movable tables and chairs, and bedding articles have low market liquidity, convenient purchase, good corrosion resistance, and low storage cost, and can be stored for a long time. Health protection articles also have high storage resistance. After a disaster breaks out, sanitation and disinfection are an indispensable part, so the importance of storage is the highest.

The reserve index is also high for food categorized by bottled water, ready-to-eat grain, radish, and other shelf-stable vegetables, convenience food, and other food security necessities. When typhoons, earthquakes, floods, and other natural disasters occur, there is likely a shortage of enough food and clean water, instant noodles, biscuits, bread, and other convenient foods. These food security necessities have the advantages of rapid collection, convenient transportation, resistance to damage (anti-fall, and anti-corrosion), and the reserve value is high, so the reserve is of high importance.

In the third place are sanitary necessities such as cleaning and disinfection products and daily necessities, mainly including sterilized alcohol, simple toilets, toilet paper, toothbrushes, laundry detergent, wet paper towels, towels, etc. These are the basic daily necessities for people’s daily lives for survival and maintaining personal hygiene and health, and protect them from diseases. In addition, sanitary necessities generally have a long shelf life, are relatively storable, and are relatively inexpensive to store, thus having a high storage necessity.

The fourth and fifth places in the reserve index are medical and pharmaceutical necessities such as first-aid medical kits and clothing protection necessities such as uniforms and warm clothes. Medical and pharmaceutical necessities play the role of timely prevention, control, and treatment of unexpected diseases or injuries, and prevent life damage caused by failure to seek medical treatment in time due to special emergencies. Appropriate storage can alleviate the drug demand of special people and special events, and clothing plays an important role as the defense line of the outer skin.

The lowest storage index is aquatic products, edible fungi, fruits, green leafy vegetables, and other non-staple foods. These have high nutritional value, but their short shelf life and high storage requirements (special storage conditions, high storage cost, and high storage risk) make them less suitable for large-scale reserves.

According to the principle of 3 s mathematical statistics, the standard deviation of 1–2 times the average value of different varieties’ reserve index is selected. In this study, the reserve grades of different types of daily necessities are classified as managed, which are: must reserve, should reserve, consider reserve, and not suitable for reserve. The specific classification is shown in Table 2.

**Table 2:** Emergency necessities reserve level.

Category	Subdivision category	Suggested level
Clothing security category	Daily wear	Consider reserves
	Warm clothing	Consider reserves
Food security category	Processed food	Should be reserved
	Instant food	Should be reserved
	Bottled drinking water	Should be reserved
	Edible oil	Consider reserves
	Salt	Consider reserves
	Leafy greens and other storage-intolerant vegetables	Not suitable for storage
	Storage-resistant vegetables such as turnips and potatoes	Should be reserved
	Mushrooms	Not suitable for storage
	Fruits	Not suitable for storage
	Livestock meat	Consider reserves
	Aquatic products	Not suitable for storage
	Eggs	Consider reserves
	Liquid milk	Consider reserves
	Milk powder	Consider reserves
Temporary accommodation category	12 m^2^ single tent	Must be reserved
	Bedding	Must be reserved
	Folding bed	Must be reserved
	Pillows	Must be reserved
	Mobile table and chairs	Must be reserved
Hygiene products	Cleaning and disinfecting products	Should be reserved
	Daily life supplies	Should be reserved
	Hygiene and security supplies	Must be reserved
Medical drugs	First aid medical kits	Consider reserves

### 3.2 Analysis of influencing factors of daily necessities reserve

Daily necessities are closely related to people’s daily life. The effective management of daily necessities reserves requires optimizing the role of policy guidance and market mechanism, while also strengthening resource integration to avoid redundant reserves and realizing resource sharing. This approach can better enhance the emergency response capability of daily necessities. To better meet the emergency needs, this paper focuses on the principle of “prevention first, combining peacetime with disasters; government-led and social participation; under the basic principle of hierarchical management and integration of resources (33).” From the perspective of ensuring the most basic living needs of urban residents, the main indicators that affect the reserve quantity of daily necessities include:

Size of regional resident population. The larger the regional population base is, the more people will be affected during crisis and the greater the consumption of daily necessities will be. Under emergency and crisis, local governments usually make detailed material reserve plans according to the size of the resident population, medical resources, and production capacity. Therefore, the size of the regional resident population is an important basis for the reference of daily necessities reserves.

The proportion of urban residents in the region. In areas with a high proportion of rural residents, the self-sufficiency rate of agricultural and sideline products and other necessities is relatively high. Cities with a higher proportion of urban residents have greater dependence on external supplies due to factors like logistics limitations during emergencies. When emergencies occur, there may be situations such as logistics blocking, isolation control, road closure, etc., which hinder the logistics supply, procurement, and retail of daily necessities. Therefore, such areas need to reserve more materials to deal with emergencies, so urban residents’ daily necessities are highly dependent on foreign countries.

Probability of disaster occurrence. Emergencies lead to uncertainty and interruption risk in the supply chain environment. In the emergency supply chain, mitigating such risks depends on the efficient distribution of emergency materials (34). Often, the greater the probability of regional disasters, the higher the demand for emergency materials (35), the greater the probability of mobilizing daily necessities reserves, the stronger the necessity of daily necessities reserves, and the more effective the storage of stored materials can be.

Population density. To a certain extent, population density is closely related to the urgency of demand (36). The greater the population density, the higher the urgency of the demand for necessities in an emergency, and the more reserve materials are allocated to this area.

Public budget. A larger public budgets signifies greater economic strength that can be allocated towards reserves. This reflects the government’s willingness to support public undertakings.

Regional income level. Varying income levels across regions translate to different consumption structures. In the process of storing daily necessities, it is necessary to accurately match the diversified needs of consumers. In addition to meeting the minimum use value demand, the products and services provided should also consider the diversified and differentiated needs of the people and provide differentiated living materials protection.

## 4 Results

Taking into account the above influencing factors, combined with the results of the single-variety reserve index for necessities, the key variables are then sequentially identified to measure the number of reserves of necessities. First, determine the per capita demand for various types of necessities by the “14th Five-Year Plan” for emergency material security. The food and nutrition development goals in the “China Food and Nutrition Development Program” issued by the State Council determine the per capita intake of food and nutrition. The per capita demand for clothing protection category, temporary accommodation category, hygiene supplies category, and medical drugs category materials refer to policy documents such as Classification and Coding of Emergency Supplies, Classification Catalog of Key Emergency Supplies for Emergency Protection, Suggested List of Emergency Supplies Reserve for Families in Shanghai, and Guidelines on the Standard of Reserve of Disaster Relief Supplies. The final organization is shown in Table 3.

**Table 3:** Collation of per capita demand for necessities.

Category	Subdivision category	Per capita demand
Clothing security category	Daily wear	1 set/person
	Warm clothing	1 set/person
Food security category	Processed food	1 kg/person/day
	Instant food	500 g/person/day
	Bottled drinking water	300 g/person/day
	Edible oil	25 g/person/day
	Salt	10 g/person/day
	Leafy greens and other storage-intolerant vegetables	200 g/person/day
	Storage-resistant vegetables such as turnips and potatoes	200 g/person/day
	Mushrooms	200 g/person/day
	Fruits	160 g/person/day
	Livestock meat	45 g/person/day
	Aquatic products	49 g/person/day
	Eggs	43 g/person/day
	Liquid milk	300 g/person/day
	Milk powder	300 g/person/day
Temporary accommodation category	12 m^2^ single tent	0.25 tops/person
	Mobile table and chairs	0.25 sets/person
	Folding bed	1 piece/person
	Futon	1 bed/person
	Pillow	1Pc/person
	Moisture-proof mat	1Pc/person
Hygiene products	Cleaning and disinfecting products	1 set/person
	Daily life supplies	1 set/person
	Hygiene and security supplies	0.1 set/person
Medical drugs	First aid medical kits	1 set/person

Second, the number of people affected by the disaster is determined. This study determines the affected population according to the minimum value of the number of people in need of emergency relocation or emergency living assistance under the emergency response of the Shanghai Disaster Relief Emergency Plan, and the minimum number of people in need of emergency living assistance under the level I emergency response is 500,000, followed by 300,000 for level II, 100,000 for level III, and 30,000 for level IV. Based on these figures, the reserve quantity of necessities of life under the emergency response at all levels is calculated. The reason for this is that the above-mentioned emergency supplies for the living category are characterized by a large number of demand categories but a small demand, are not consumables, and can be used repeatedly. Compared to dietary protection supplies, the emergency supplies for the living category have longer storage and re-placement cycle. Therefore, the specific emergency supply reserve can be calculated according to the number of people covered in the emergency response plan.

Finally, according to the intensity and coverage of different disaster situations, the dietary protection category of supplies is calculated as the amount to guarantee a 3-day and 7-day supply, respectively. Other categories of materials can be used for a long time without considering the number of days of supply, and the number of days of supply is set to 1. The result of the calculation of the total amount of emergency staples and supplementary foods in Shanghai for all levels and categories is shown in Table 4.

**Table 4:** Stockpile of materials in the category of dietary protection under various levels of emergency response (unit: tons).

		Level I emergency response		Level II emergency response		Level III emergency response		Level IV emergency response	
Category	Subdivision category	For 3 Days	For 7 Days	For 3 Days	For 7 Days	For 3 Days	For 7 Days	For 3 Days	For 7 Days
Food security category	Drinking water	1,500	3,500	900	2,100	300	700	90	210
	Processed food	750	1750	450	1,050	150	350	45	105
	Instant food	450	1,050	270	630	90	210	27	63
	Edible oil	37.5	87.5	22.5	52.5	7.5	17.5	2.25	5.25
	Salt	15	35	9	21	3	7	0.9	2.1
	Storable vegetables	300	700	180	420	60	140	18	42
	Livestock meat	67.5	157.5	40.5	94.5	13.5	31.5	4.05	9.45
	Eggs	64.5	150.5	38.7	90.3	12.9	30.1	3.87	9.03
	Milk powder	450	1,050	270	630	90	210	27	63
	Liquid milk	450	1,050	270	630	90	210	27	63

As can be seen from Table 4, among the necessities in the category of dietary security, packaged food and bottled water have the largest quantity of reserves. This is due to two factors: firstly, the high demand—an adult needs to consume 250 g of staple food and 1 kg of water a day as a basic need – with inelastic demand that remain consistent even during emergencies; and secondly, it is concluded from Table 2 that the reserves of these abovementioned necessities have a high priority, and the difficulty of stock-piling them is relatively low. Fresh vegetables, including green leafy vegetables, edible mushrooms, and other perishable vegetables, are not easy to reserve. Vegetable reserves primarily consist of radishes, potatoes, and other vegetables with longer storage life, which coincides with the reality of the emergency supply. The reserve quantities of livestock meat and eggs is not very different, reflecting the difficulty and the cost of maintaining the reserve, and can be considered to mix the reserve. For milk, while liquid milk is more popular than milk powder under normal circumstances, milk powder offers the same protein supply efficiency in a much small volume and a longer shelf life. Therefore, a certain amount of milk powder should be included in reserves, which is consistent with the real-world scenarios. Similarly, for other categories of materials can be used for a long period without considering the number of days of supply, the number of days of supply is set to 1. The results of expanding the reserve of materials from the category of dietary protection to the categories of clothing protection, temporary accommodation, sanitary supplies, and medical drugs under various levels of emergency response are shown in Table 5.

**Table 5:** Stockpiles of clothing protection, temporary accommodation, hygiene supplies and medical drugs under various levels of emergency response.

		Level I emergency response	Level II emergency response	Level III emergency response	Level IV emergency response	
Category	Subdivision category	Reserve				Unit
Clothing security category	Daily wear	500,000	300,000	100,000	30,000	Set
	Warm clothing	500,000	300,000	100,000	30,000	Set
Temporary accommodation category	12 m^2^ single tent	125,000	75,000	25,000	7,500	Set
	Mobile table and chairs	125,000	75,000	25,000	7,500	Set
	Folding bed	500,000	300,000	100,000	30,000	Sheet
	Futon	500,000	300,000	100,000	30,000	Piece
	Pillow	500,000	300,000	100,000	30,000	Piece
	Moisture-proof mat	500,000	300,000	100,000	30,000	Piece
Hygiene products	Cleaning and disinfecting products	500,000	300,000	100,000	30,000	Set
	Daily life supplies	500,000	300,000	100,000	30,000	Set
	Hygiene and security supplies	50,000	30,000	10,000	3,000	Set
Medical drugs	First aid medical kits	500,000	300,000	100,000	30,000	Piece

## 5 Algorithmic test and material distribution

### 5.1 Algorithmic test

The number of people affected by floods is a key variable in the calculation of the number of reserves of necessities, the more the number of people affected by floods, the higher the demand for necessities. Therefore, this paper takes the affected population as the basis for the test of the amount of reserves of necessities.

The number of people affected by floods in Shanghai is estimated by the percentage of flood victims in the whole country, and the estimated number of affected people is compared with the number of relief population in the emergency response plan to check whether it can be covered by the number of relief population in the emergency response plan given in this paper, to verify the reliability of the study. According to the data from the National Bureau of Statistics, the number of people affected by natural disasters in 2021 is 107 million, of which 59.01 million people are affected by floods, and the number of people affected by floods accounts for 55.1% of the number of people affected by natural disasters in the country.


(4) $$\begin{array}{c} \mathrm{Percentage}\;\mathrm{of}\;\mathrm{national}\;\mathrm{flooding}=\frac{\mathrm{national}\;\mathrm{flooding}}{\mathrm{national}\;\mathrm{natural}\;\mathrm{disaster}}\;\\ =\frac{0.0591}{1.07}=55.1\%\# \end{array}$$

In 2021, the total population of Shanghai is 24.894 million. The number of people affected by natural disasters in Shanghai is 734,000 people. According to the above Eq. (4) to get the national flood disaster proportion of the projected number of people affected by floods in Shanghai is 404,400 people.


(5) $$\begin{array}{l} \mathrm{The}\;\mathrm{total}\;\mathrm{population}\;\mathrm{affected}\;by\;\mathrm{natural}\\ \mathrm{disasters}\;\mathrm{in}\;\mathrm{Shanghai}\times \;\mathrm{the}\;\mathrm{proportion}\;\mathrm{of}\;\mathrm{national}\;\mathrm{flooding}\\ =73.4\times 55.1\%=404,400\;\mathrm{people} \end{array}$$

The minimum number of people who need to be rescued in the level I emergency response to natural disasters in Shanghai is 500,000 people, which can cover the projected value of 404,400 people derived from Eq. (5), so the number of reserves under the level I emergency response calculated in this paper can satisfy the emergency demand for necessities in Shanghai in different scenarios, and the results are informative.

### 5.2 Construction of a responsive material deployment system

#### 5.2.1 Strengthen the monitoring of supply and demand of daily necessities

In case of emergency, continuously track and monitor the output, stock, and consumption of important emergency necessities, and monitor and collect the demand for daily necessities through multiple channels. Based on factors such as the severity, development trend, and influence range of disasters, the supply and demand of daily necessities are analyzed and predicted using expert analysis, auxiliary decision-making, consultation, and judgment, to accurately guide the preset and financing preparation of daily necessities and transportation capacity.

#### 5.2.2 Improve cross-sectoral and cross-regional redeployment mechanisms

Refine and improve the response procedures for the deployment of daily necessities, and standardize the management of demand submission, allocation approval, instruction issuance, capacity raising, material transportation, reception, and use. At the same time, the centralized management and unified dispatch of daily necessities during emergency response can be strengthened, and daily necessities command and dispatch drills can be carried out regularly to improve the ability of material allocation and coordinated operations.

#### 5.2.3 Improve material transportation capacity

Strengthen the emergency transport capacity reserve. Coordinate passenger transport, freight transport, heavy cargo, cold chain distribution, port shipping, and other emergency transport capacity reserves, and establish an emergency transport vehicle and driver information ledger to do dynamic management. By mobilizing state-owned transport enterprises as the “mopping force” of the urban supply chain, logistics enterprises are selected to join the transportation in the city, and the business is carried out according to the time and place, and the regional multimodal transport is refined, the shared logistics model is promoted, and the application of technologies such as drone transportation is explored to improve the distribution capacity of daily necessities (37).

#### 5.2.4 Strengthen the coordinated support of transportation

Improve the emergency transportation linkage mechanism, including the choice of various materials transportation modes, unified vehicle dispatching plan, and do a good job in emergency transportation guarantees such as emergency transportation of daily necessities, capacity dispatching, and timely repair (38). In case of emergency, a “green passage” for the transportation of daily necessities is set up to realize rapid passage, thus ensuring the priority arrangement and scheduling of daily necessities. Strengthen the coordination with the reserves of daily necessities in adjacent areas, establish channels for material transfer, promote the sharing and linkage of daily necessities across regions, and enhance the ability of mutual assistance and support. Actively mobilize logistics enterprises, enterprises and institutions, social organizations, and volunteers to participate in the delivery, receipt, and distribution of materials, and comprehensively improve the ability of material distribution.

Shanghai’s largest material storage warehouse is located in Minhang District, a food material guarantee warehouse established by the government. Shanghai Wusong International Logistics Park, located in Baoshan District, Shanghai, is an international large-scale warehouse. The two areas are located near the city center, with a large area and dense population, which is convenient to support other areas as logistics nodes while rescuing the local area. We can choose the area where the two warehouses are located as the supply point for dispatching in the city to allocate daily necessities to other regions. There is also the Pudong New Area, where the economy is developed and the transportation network is relatively perfect. The total transportation distance to other districts and counties in the community is short, the city’s comprehensive support ability is strong, and the logistics cost control options are wide (39). As a densely populated city with a large number of urban areas, Shanghai has a developed traffic network, but it is easy to cause traffic jams. It is also important to take the actual situation of highways into account and plan according to local conditions.

Based on comprehensively improving the above points, taking Shanghai as an example, under the background of flood disasters, we can use the distribution method of daily necessities supply points with the optimal supply and demand distribution model as the core. This method aims at minimizing the total distance to meet the demand. It considers the constraint of Shanghai market reserve points on the guaranteed supply of daily necessities to the original demand points to optimize the distribution of market reserve points for shelter supply. At the same time, based on the real road network, the reliability of road sections is evaluated by using the “degree,” “square agglomeration coefficient” and “daily traffic volume of road design” (40), and the road transit time is evaluated by considering the wading speed. With the goal of “shortest path travel time” and “highest path reliability,” the optimal distribution path evaluation model between stations is constructed, to compare various algorithms to optimize the distribution path.

## 6 Discussion

Taking Shanghai as an example, this paper calculates the variety and quantity of urban necessities reserve. Based on the definition and connotation of urban necessities, the model of the urban necessities reserve index is established. It considers four aspects: market liquidity, production cycle, storage durability, and reserve cost. The subjective and objective combination method of the Delphi method and the entropy weight method is used to determine the weight of evaluation indicators, which solves the problems of subjective interference and the great difference between the results of the objective weighting method and reality. This approach provides a more practical way to categorize emergency supplies: necessities of life that must be stockpiled, should be stockpiled, should be considered for stockpiling, and are not suitable for stockpiling. Furthermore, the model clarify the scientific basis for determining “what to stock” and lays the scientific basis for “how much to stockpile” based on a model for emergency stockpiling of necessities of life. The local government needs to increase the financial guarantee for emergency materials reserves, provide certain policy support and financial subsidies for emergency materials production enterprises and daily necessities production enterprises (41), and pay attention to a stable supply of daily necessities for different groups of people during emergencies. Meanwhile, after settling on “what to store” and “how much to store,” we should think deeply about “how to store” and “who will store.” We should also establish and improve the emergency storage and transportation mechanism, ensure smooth transportation, increase cooperation between the government and storage enterprises, entrust enterprises to produce, purchase, store, transport and dynamically track the necessary emergency necessities, ensure the use efficiency and allocation speed of materials, and provide stable and reliable inventory and transportation support for emergency work.

Based on the above analysis, this paper puts forward the following suggestions:

First, strengthen monitoring and early warning. (1) Enhance urban risk monitoring: systematically arrange and categorize the demand for emergency materials for natural disasters, accidents, disasters, social security and other emergencies according to Shanghai’s urban safety risks and potential hazards, improve the reserve layout, and optimize the reserve scale and structure of emergency necessities; (2) Strengthen market monitoring and supervision: strengthen data sharing, analysis and judgment, establish a linkage mechanism for store managers in surrounding supermarkets, implement dynamic monitoring and timely track and guide scheduling, to provide decision support for timely response to emergencies.

Second, revise and optimize the catalog of daily necessities according to the guiding catalog for storage stipulated by the local government. This will help reduce the storage cost and improve the storage capacity. Implement a multi-level management system for the essential reserves, recommended reserves, the reserves that should be considered, and the reserves of daily necessities that are not suitable for storage to improve the efficiency of emergency reserves.

Third, scientifically plan emergency material reserves. Consider factors like the proportion of urban residents, the number of permanent residents, the probability of disasters, the population density, the public budget, and the per capita disposable income of the region. This will ensure a scientifically and rational plan for the reserve of emergency materials and minimize the waste of reserve materials.

Fourth, improve personnel security, financial security and technical security. First, increase financial investment and allocate a reasonable budget for emergency expenditure, and ensure that emergency funds are in place in time. Second, establish an integrated platform for local risk early warning that enables information sharing. Leverage information technology to understand changes in the consumption demand of public necessities, and establish and improve the database of daily necessities. This allows for timely adjustments to the varieties of daily necessities monitored.

The deficiency of this paper lies in the factors that affect the single variety reserve index and the main indicators that affect the quantity of daily necessities reserve, which can be further enriched and improved. The influencing factors listed in this paper come from the existing literature and research contents, and the more detailed screening of influencing factor indicators is necessary for real-world application. If it is to be applied to practical work, the index system needs to be further improved. Additionally, the research primarily focuses on Shanghai. In future practical work, we will further enrich the width of the evaluation index system by consulting the opinions of staff experts and scholars who have worked in the government extensively, and try to expand the research scope to other cities and countries to deepen the design of logistics models for the distribution of daily necessities.

## Data availability statement

The original contributions presented in the study are included in the article/supplementary material, further inquiries can be directed to the corresponding author.

## Author contributions

QJ: Conceptualization, Formal analysis, Funding acquisition, Methodology, Project administration, Supervision, Validation, Writing – review & editing. XJ: Conceptualization, Data curation, Formal analysis, Methodology, Project administration, Writing – original draft, Writing – review & editing. ZR: Data curation, Software, Writing – original draft.
